# Aflatoxin awareness and Aflasafe adoption potential of Nigerian smallholder maize farmers: OPEN ACCESS

**DOI:** 10.3920/WMJ2018.2345

**Published:** 2018-08-14

**Authors:** A.M. Johnson, J.R. Fulton, T. Abdoulaye, B. Ayedun, N.J.O. Widmar, A. Akande, R. Bandyopadhyay, V. Manyong

**Affiliations:** 1 Department of Agricultural Economics, Purdue University, 403 West State Street, West Lafayette, IN 47907, USA; 2 International Institute of Tropical Agriculture (IITA), PMB 5320, Oyo Road, Idi-Oshe, Ibadan, Nigeria; 3 IITA, PMB 82, Garki GPO, Kubwa, Abuja, Nigeria; 4 IITA, Plot No. 25, Mikocheni Light Industrial Area, Mwenge - Coca-cola Road, Mikocheni B, P.O. Box 34441, Dar es Salaam

**Keywords:** mycotoxin, biological control, atoxigenic strain, technology uptake, Nigeria

## Abstract

Aflatoxin is a potent mycotoxin that can cause cancer and death and is associated with stunted growth. Prevalence of aflatoxin is widespread in Africa negatively impacting health and trade. Aflasafe is a biological control product that can be applied to maize or groundnut fields to reduce aflatoxin contamination. This study examines the levels of aflatoxin and Aflasafe awareness and understanding among smallholder maize farmers in Nigeria. In addition, the factors affecting Aflasafe purchase patterns and sustained usage over multiple growing seasons by farmers were evaluated. In-person surveys of 902 Nigerian smallholder farmers were conducted during October and November of 2016. This work contributes to the existing literature by documenting awareness levels of aflatoxin and use of Aflasafe as a control in Nigeria. Results suggest that the level of awareness of aflatoxin was very high in states where Aflasafe was promoted as an intervention for aflatoxin management. In Kaduna state, the region with the longest intervention, there was a consistent increase in the usage of Aflasafe since its introduction in 2010. Furthermore, farmers who purchase Aflasafe bundled (combined) with other inputs were more likely to persist in using the product. Education was found to significantly and positively impact continued usage of Aflasafe. Continued interventions, promotion and general education of the public are recommended for increased awareness, trial, and adoption of Aflasafe in Nigeria.

## 1. Introduction

Maize and groundnuts are significant sources of human food, animal feed and income in sub-Saharan Africa. Maize is Africa’s most important food crop grown on nearly 30 million hectares of land and supporting over 300 million people on the continent (Fisher *et al.,* 2015). It is an important income generating food staple in Nigeria and its importance has been increasing over the years (Abdoulaye *et al.,* 2018). Nigeria is the largest maize and groundnut producer in West Africa (FAOSTAT, 2017). Maize and groundnuts are particularly prone to aflatoxin contamination (Bandyopadhyay *et al.*, 2016; Liu and Wu, 2010). In their paper, Ogara *et al.* (2017) reported that 47% of their samples of Nigerian maize exceeded the European Union limit of 4 µg/kg for aflatoxins in food. Aflatoxin is a highly toxic metabolite produced by members of *Aspergillus* section *Flavi –* primarily *Aspergillus flavus* and *Aspergillus parasiticus –* commonly found in soils and on grain and legume crops (Bandyopadhyay *et al.*, 2016; Williams *et al.*, 2004).

Chronic aflatoxin ingestion has been linked to liver cancer, immune-system suppression, growth retardation, and more rapid progression of HIV/AIDS (Gong *et al.*, 2002; Turner *et al.*, 2003; Williams *et al.*,^ 2004^). Furthermore, individuals already infected with Hepatitis B are at higher risk of liver cancer when co-exposed to aflatoxin B_1_ (Groopman *et al.*,^2008^). Acute aflatoxicosis can cause rapid death from liver failure, as exemplified by the death of 125 Kenyans in 2004 (Azziz-Baumgartner *et al.,* 2005). Beyond the human health impacts of directly consuming aflatoxin-contaminated food, there are also negative impacts on farm animals consuming aflatoxin-contaminated feed, which lead to a reduced growth rate and productivity of the animals (Bryden, 2012) and attendant loss of income by poultry, dairy and fish industries. Furthermore, aflatoxin residues have been found in dairy, meat, and poultry products originating from animals fed aflatoxin-contaminated feed (Iqbal *et al.*, 2014; Keyl and Booth, 1971).

The aflatoxin contamination in food can only be accurately quantified with laboratory testing. Hoffmann *et al.* (2013) found that retail prices of maize in Eastern Kenya were negatively correlated with the number of discoloured kernels in the grain sample and suggested that the discolouration of kernels (which is observable) may serve as a proxy to buyers of the level of contamination (which is unobservable) in maize. Along maize and groundnuts value chains, there is generally a low level of awareness about aflatoxin and its consequences. Ezekiel *et al.* (2013) found that only 15% of consumers in five Nigerian states (Lagos, Ogun, Oyo, Niger, and Kaduna) were aware that groundnut (peanut) cakes could be aflatoxin-contaminated. Also, De Groote *et al.* (2016) discovered, from their study in Kenya, that 64% of consumers interviewed were aware of aflatoxin, but only 16% understood its health risks. In a review article (worldwide), Ragona (2016) found very scarce evidence of the level of understanding of aflatoxin by consumers.

Several pre-and post-harvest methods have been recommended for aflatoxin mitigation (Udomkun *et al.*, 2017; Wagacha and Muthomi, 2008). Biological control is one of the most promising and cost-effective methods to reduce aflatoxin in maize and groundnut (Bandyopadhyay *et al.,* 2016; Dorner, 2004; Wu and Khlangwiset, 2010). Aflasafe is a novel biological control product composed of native strains of *A. flavus* that do not produce aflatoxin (Bandyopadhyay *et al.*, 2016). When Aflasafe is introduced in a field, the non-toxic strains out-compete the pre-existing toxic strains through a process known as competitive exclusion (Atehnkeng *et al.*, 2014). Reductions in aflatoxin contamination of >80% have been documented in fields treated with Aflasafe compared to untreated fields (Bandyopadhyay *et al.*, 2016). The benefits of Aflasafe continue during crop storage because the non-toxic *A. flavus* remains on maize, still competing with the toxic *A. flavus* strains that would otherwise increase aflatoxin levels during storage (Atehnkeng *et al.*, 2014). Unique Aflasafe products are registered for commercial use in several African nations including Nigeria (Bandyopadhyay *et al.*, 2016).

Since the 2014 cropping season, the AgResults Nigeria Aflasafe pilot project is facilitating adoption of Aflasafe by farmers in the country. Through the AgResults intervention explained below, a group of agribusiness companies are enrolled in the pilot project to provide Aflasafe and other inputs on credit, and training to participating farmers. In addition, these companies purchase the Aflasafe-treated maize and aggregate it for sale to food and feed processors at a premium. Maize samples are collected from aggregated lots by AgResults project personnel for analysis to determine whether Aflasafe had been applied to the crop. Previous research had determined that maize that has a high preponderance of Aflasafe strains (i.e. *Aspergillus* strains constituting Aflasafe product) have low aflatoxin concentration (Atehnkeng *et al.* 2014). The pilot project pays an incentive of $18.75 per ton of maize with acceptable levels of Aflasafe strains (Bandyopadhyay *et al.*, 2016).

The regular continued use of Aflasafe by smallholder farmers is important for its commercialisation and contribution to the production of a safe food supply, economic development and poverty alleviation. Aflasafe is a relatively unknown product for most farmers as well as others along the maize value chain in Nigeria. Longstanding marketing theory identifies awareness as the first stage in the process of new product adoption, while trial, adoption and confirmation are the final stages. The level of, and factors affecting awareness of aflatoxin and Aflasafe among small-holder farmers is not known. In addition, there are few published works on the use and adoption of Aflasafe in Africa. Therefore, the objectives of this research were to (1) assess the extent of awareness and understanding of aflatoxin and Aflasafe among smallholder maize farmers in Nigeria, and (2) to identify factors affecting Aflasafe purchase patterns and sustained Aflasafe usage over multiple growing seasons by farmers. Information generated from this research can guide approaches for enhancing commercialisation and adoption of Aflasafe for food security and income generation in Nigeria.

## 2. Materials and methods

### Selection of study sites

**Figure 1:**
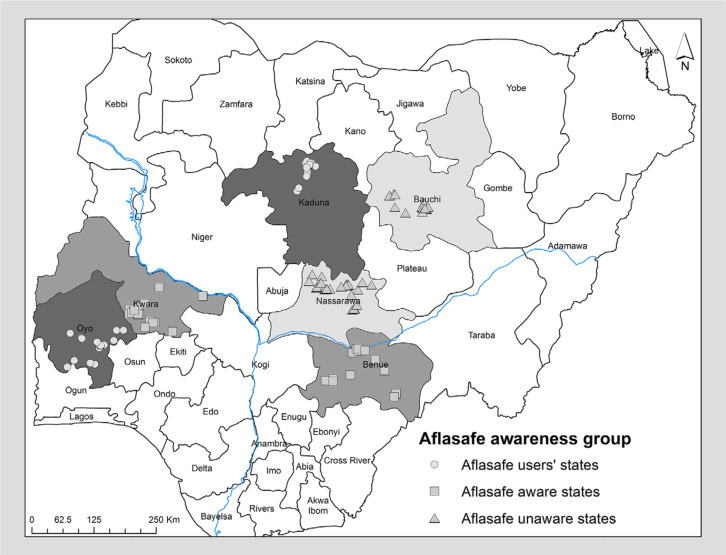
Map of Nigeria showing three clusters of states where maize farmers were surveyed.

Using historical information on areas where Aflasafe adoption was promoted by the AgResults project, states with contrasting characteristics were identified for this study. Respondents for surveys were selected from two states each in three clusters of states based on the level of experience farmers had with Aflasafe: Cluster A, those who had used Aflasafe in the past; Cluster B, those who were aware of Aflasafe but had not used the product, and Cluster C, those who were not aware of Aflasafe (Figure 1). Farmers in the states of Kaduna and Oyo had experience of using Aflasafe due to longer exposure beginning with demonstration trials prior to 2014 and continued through the intervention from the AgResults pilot project. In the states of Kwara and Benue, farmers were aware of the existence of Aflasafe, but up to the time of the study had no direct experience, access and application of the product. Finally, farmers in the states of Nassarawa and Bauchi had no direct knowledge of Aflasafe.

### Data collection

Primary data using in-person surveys were collected during the period of October to November 2016 in the six Nigerian states highlighted in Figure 1. Data collected from each respondent include demographics, awareness about aflatoxin and Aflasafe, use of Aflasafe, crops grown and use of other inputs, such as fertiliser, and seeds. A total of 150 farmer responses in each state were targeted. In each state, 10 villages were randomly selected from the AgResults Nigeria pilot project database or with assistance from extension services (for states with no project sites). On average, 15 farmers were randomly selected in each village for the survey. Due to the small number of farmers with experience of using Aflasafe in Oyo state, it was necessary to oversample in Kaduna state. Out of 902 farmers surveyed, 320 were current or former Aflasafe users (Cluster A), 285 were aware of Aflasafe but had never used it (Cluster B), and 297 were not aware of Aflasafe (Cluster C).

The surveys received approvals from review boards of the authors’ respective institutions. Surveys were administered by enumerators who received verbal consent from respondents before proceeding with surveys. All survey enumerators had language skills proficient enough to communicate with the farmers in local dialects. They also all had education levels equal to or exceeding a bachelor’s degree and were trained on the survey protocols during a two-day workshop in September 2016.

### Data analysis

Data were analysed via cross-tabulations mainly for objective 1 (assess the extent of awareness and understanding of aflatoxin and Aflasafe) and a *logit* regression for objective 2 (to identify factors affecting Aflasafe purchase patterns and sustained Aflasafe usage). *Logit* regression is a standard tool used to predict the outcome of binary dependent variables. *Logit* regressions assume that the probability of the dependent variable, y, taking a value of unity (Prob(y=1)) can be estimated with a logistic function (Wooldridge, 2013, pp. 585).

$$\text{Prob(y=1|x) = Gβx}\prime )={\text{e}}^{\wedge }\left(\text{βx}\prime \right)/\left(1+{\text{e}}^{\wedge }\left(\text{βx}\prime \right)\right),\text{y }\in (0,1)\;$$ 1

In Equation 1, y takes on the value of 1 when a given outcome is observed and a value 0 when it is not. Specifically, y=1 when a farmer continued using Aflasafe in the 2016 growing season and y=0 when a farmer stopped using Aflasafe by the 2016 growing season. e is Euler’s number. x is a matrix vector of independent (or explanatory) variables. β is a vector of coefficient parameters to be estimated using maximum likelihood estimation. The logit regression was run in STATA 14.0 (StataCorp LLC, College Station, TX, USA).

## 3. Results

### Demographics of respondents

The demographics of the farmers in this study is summarised in Table 1. The average household head in the survey was relatively young (44.1 years) but well experienced in farming as they spent almost one-half of their lives in farming. About 13 people lived in each household. Household size is relatively large compared to the national average of Nigeria of 5.9 persons in rural areas (NBS, 2016). The respondents used nearly one-half of the cultivated land for growing maize denoting the importance of the crop. Furthermore, 106 respondents (11.8% of sample) were female.

**Table 1:** Demographic characteristics of respondents (n=902) who participated in the survey to assess awareness of aflatoxin and Aflasafe.

Characteristics	Mean	Median	SD^1^
Age of farmer (number in years)	44.1	42.0	12.6
Household size (number of people)	12.6	10.0	10.0
Education level [Highest years of education attained by any household member] (number in years)	13.0	14.0	3.7
Farming experience (number in years)	21.5	20.0	12.0
Total cultivated land (hectares)	7.8	5.0	8.8
Land for maize cultivation (hectares)	3.8	2.5	4.7

^1^ SD = standard deviation.

### Awareness

**Figure 2:**
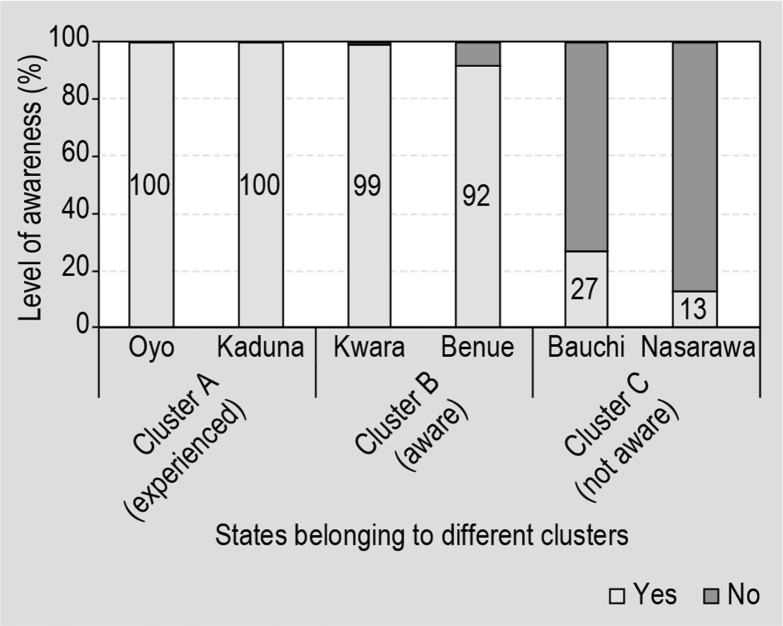
Level of awareness of aflatoxins among Nigerian farmers in three clusters of states, each with contrasting experiences of Aflasafe use.

The extent of awareness differed between the clusters (Figure 2). In Oyo and Kaduna states where farmers had experience with Aflasafe, 100% of the surveyed farmers were aware of aflatoxin, as expected. In the states of Kwara and Benue, selected for general awareness of Aflasafe, 1% and 8% of surveyed farmers, respectively, had not heard of aflatoxin. Product awareness is gradual, with some individuals within a group becoming aware sooner than others. Therefore, it is not surprising that some farmers are still not aware of aflatoxin in these two states. In Bauchi and Nassarawa, the states selected for not being aware of Aflasafe (cluster 3), already 27 and 13%, respectively, of farmers surveyed are aware of aflatoxin.

**Figure 3:**
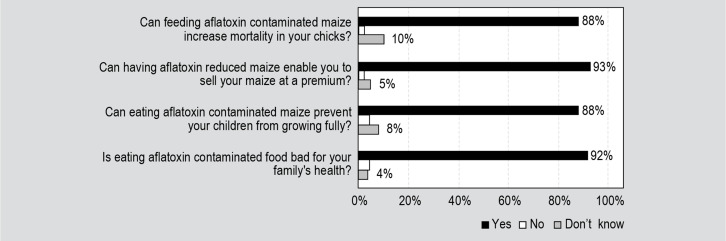
Perceptions about aflatoxin among surveyed Nigerian farmers who are aware of aflatoxins (n=648).

The 648 farmers who had heard of aflatoxin were asked four questions about the impacts they believe aflatoxin have on human health, poultry health, and maize pricing (Figure 3). Among these 648 farmers, 88% respondents had heard that aflatoxin stunts child growth and 92% believed aflatoxin is bad for their family’s health. About the same number of farmers who believed that aflatoxin inhibits growth in human children also believed aflatoxin increases mortality in chicks. More than 90% of the farmers who responded claimed that aflatoxin-reduced maize could sell at a price premium.

**Figure 4:**
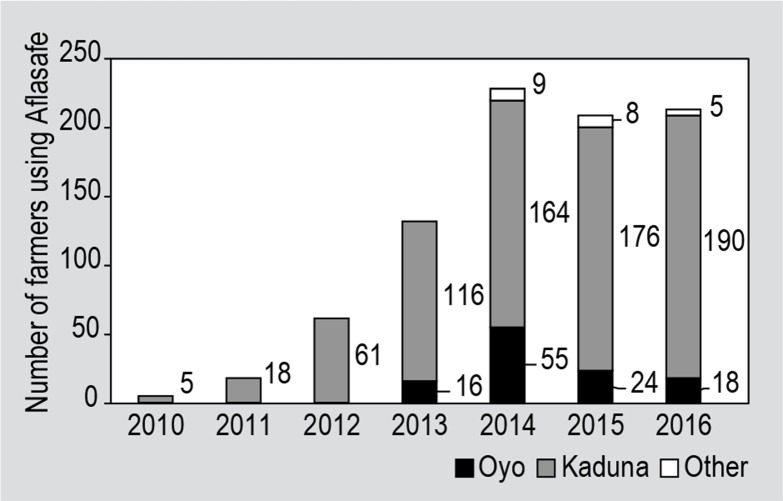
Number of surveyed farmers who used Aflasafe in Oyo, Kaduna, and other states from 2010 to 2016.

The numbers of surveyed farmers using Aflasafe in each year are shown in Figure 4. Most of these farmers were from Oyo and Kaduna state with a small number from other states. During the period of 2011 to 2016 the number of farmers in the sample who used Aflasafe in Kaduna State increased every year. In contrast, the number of those who used Aflasafe among surveyed farmers in Oyo State increased between 2012 and 2014 and then decreased.

**Figure 5:**
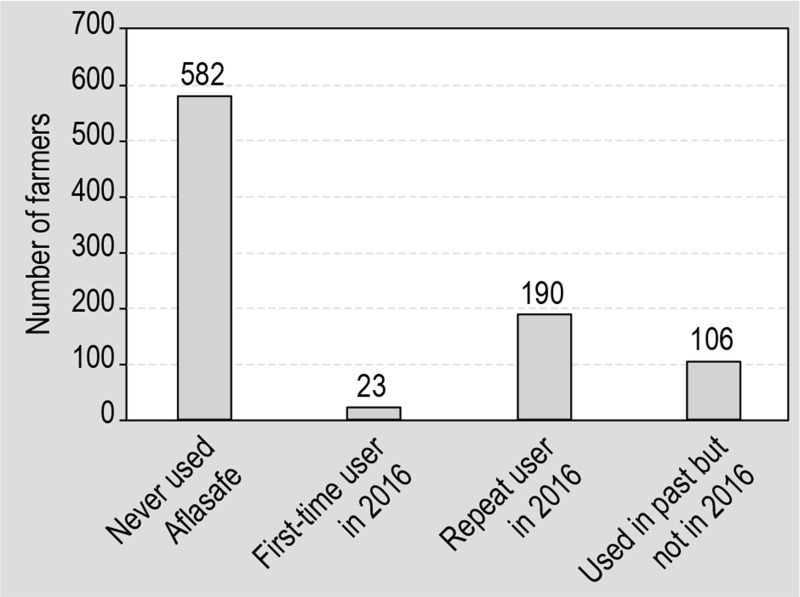
Experience of Aflasafe usage among surveyed farmers in six states in Nigeria in 2016 (n=901).

The reported levels of farmer experience having used Aflasafe are shown in Figure 5. More than half (64%) of the study participants have never used Aflasafe. From the 36% who had ever used Aflasafe, a small percentage (7%) were 2016 first time users, 60% were repeated users and 33% had used Aflasafe in the past but were not currently using it due to various reasons.

To explore the factors that drive farmers’ decisions about whether to continue using Aflasafe over multiple growing seasons, the 296 farmers with experience using Aflasafe before 2016 (last two columns of Figure 5) were examined. These farmers were divided into clusters; Cluster 1 (Awareness and Experience group) and Cluster 2 (Awareness Only group); due to spill-over effect, some members of Cluster 2 that were supposed only to be aware of Aflasafe, had started using it. Selected summary statistics for these clusters are shown in Table 2. In this sub-sample, the percentage of farmers still using Aflasafe (in 2016) was much higher in Cluster 1 (66.9%) than in Cluster 2 (13.3%). The average price paid for 10 kg of Aflasafe was the highest in Cluster 1 (at ₦3,344 compared to ₦2,144 for Cluster 2) and it was with lowest standard deviation of ₦326.

**Table 2:** Summary descriptive statistics among surveyed farmers in cluster A and B who have used Aflasafe prior to 2016 (n=296).

Clusters	n	# of farmers still using Aflasafe in 2016	# of farmers who purchased Aflasafe bundled^1^	# of farmers with formal education	Mean price paid (per 10 kg Aflasafe)^3^	Standard deviation of price^3^
Cluster A	281	66.9% (188)^2^	49.8% (140)	76.2% (214)	₦ 3,344	₦ 326
Cluster B	15	13.3% (2)	13.3% (2)	86.7% (13)	₦ 2,144	₦ 1,631

^1^ Aflasafe Bundled refers to farmers purchasing Aflasafe combined with other inputs that include credit, fertiliser, herbicide, and improved seed from their participating agribusiness company.

^2^ Numbers in parentheses are the reported frequency in each column as a percentage of the given group’s n.

^3^ 1 USD = 315 Naira (official rate in Nigeria in September-October 2016).

Almost half (49.8%) of farmers in Cluster 1 purchased Aflasafe bundled with other inputs that include credit, fertiliser, herbicide, and improved seed from their participating agribusiness company. Bundling was much less common in Cluster 2.

To more clearly identify and quantify the economic drivers of farmer persistence in using Aflasafe, a logit regression was performed as outlined in Equation 1. The results are reported in Table 3. The number of observations (n=296) for this logit regression represents all farmers who had experience using Aflasafe before 2016 (Table 3). These farmers are all from the states of Kaduna, Oyo, Kwara, and Benue. The two possible outcomes for the dependent variable of this regression were that a farmer continued using Aflasafe in 2016 (y=1) or discontinued using Aflasafe in 2016 (y=0).

**Table 3:** Results of logit regression on surveyed farmers’ persistence in using Aflasafe.

Variable name	Description^1^	Beta coefficient^1^	Average marginal effect^1,2^
Oyo	1 = farms in Oyo State	-2.762***	-0.388***
		(7.22)	(11.13)
Kwara	1 = farms in Kwara State	-3.367***	-0.473***
		(4.14)	(4.55)
Formal education	1 = received formal education	0.682*	0.096*
		(1.76)	(1.78)
Bundled	1 = purchased Aflasafe bundled with other inputs	0.907***	0.127***
		(2.67)	(2.73)
Child growth	1 = believes aflatoxin consumption reduces child growth	0.549	0.077
		(1.16)	(1.16)
Price premium	1 = believes aflatoxin-safe maize brings price premium	0.708	0.099
		(1.18)	(1.18)
Constant		-0.462	
		(0.72)	
Log Likelihood = -132.59; McFadden’s R^2^ = 0.3133			n=296
y = 1: used Aflasafe before 2016 and in 2016			
y = 0: used Aflasafe before 2016 but not in 2016			

^1^ ***, **, and * denote significance at the 1%, 5%, and 10% levels respectively. Numbers in parentheses are absolute values of z-scores.

^2^ Standard errors calculated using the delta method.

The variables identified and described in the first two columns of Table 3 are the inputs of the x vector (independent variables) in Equation 1. The Formal Education dummy variable measures whether or not a farmer obtained any level of formal education. Formal education is expected to positively affect persistence rates since literate farmers may be better able to understand what aflatoxin is, the adverse health effects of consuming aflatoxin-contaminated maize, and how Aflasafe mitigates that damage.

Similarly, the Bundled dummy variable captures whether a farmer purchased Aflasafe bundled with other inputs, such as improved seed or fertiliser. While Aflasafe has a neutral effect on maize yields, the value (or return) of the Aflasafe-treated crop is enhanced due to a reduction in aflatoxin in the grains. With the use of Aflasafe bundled with yield-enhancing inputs such as improved seed and fertiliser, farmers expect higher returns compared to using Aflasafe alone. Therefore, the expected sign on the estimated coefficient for this variable is positive.

Consumer perception about the health and economic consequences of aflatoxin contamination may also influence decisions about whether to persist in using Aflasafe. The Child Growth dummy variable and Maize Price Premium dummy variable are introduced to identify the impact of these factors and are expected to have positive coefficients.

The state dummy variables were included to capture remaining unobserved heterogeneity. The Oyo and Kwara dummy variables take the value of 1 if the farmer is from Oyo or Kwara and 0 otherwise. The reference variable for the state dummy variables which includes all other observations for farmers from Kaduna and the lone farmer from Benue was excluded from the regression to avoid perfect multicollinearity. Hence, the beta coefficient and average marginal effects for the Oyo and Kwara dummy variables are measured relative the left-out observations, which come mainly from Kaduna (205 observations).

The beta coefficients reported in Table 3 are the estimated value for the β vector in Equation 1. Because the logistic function is non-linear, beta coefficient estimates do not have a literal interpretation. When considering the estimated beta coefficients, the sign of the coefficient and whether it is statistically significant are of interest. The sign of a beta coefficient in a *logit* regression only indicates the directional impact of a change in the independent variable on the dependent variable. For this reason, the average marginal effect is calculated to show the average per-unit change in the dependent variable for every per-unit change in an independent variable (Wooldridge, 2013, pp. 291-292).

The average marginal effect of -0.388 for the Oyo dummy variable suggests that the surveyed farmers in Oyo State are 38.8% less likely to be still using Aflasafe in 2016 than the surveyed farmers from Kaduna State. Surveyed farmers from Kwara State are 47.3% less likely to be still using Aflasafe in 2016 than surveyed farmers from Kaduna State.

Farmers with formal education are estimated to be 9.6% more likely to persist in using Aflasafe in 2016 than farmers without formal education. Likewise, bundling Aflasafe with other inputs is estimated to increase the probability a farmer persists in using Aflasafe in 2016 by 12.7%. A farmer’s perception of the impact of aflatoxin on child growth and maize pricing is not estimated to have a statistically significant impact on the probability that the farmer persists in using Aflasafe over multiple growing seasons.

## 4. Discussion

In the process of adopting and using Aflasafe, a farmer is expected first to become aware of the aflatoxin problem and then aware of the potential solution via Aflasafe. Trial and adoption are the final stages of this process. James *et al.* (2007) found that 53.2% of farmers and 63.5% of consumers in a pooled sample from Benin, Ghana, and Togo were aware of aflatoxin in 2005. These percentages were significantly higher than the levels in 2000 (20.8% for farmers and 25.2% for consumers), due in part to a large public awareness campaign conducted from 2001-2004 (James *et al.*, 2007). Heterogeneity of respondent-reported aflatoxin awareness across states is evident in Figure 2. Awareness of aflatoxin ranged from as high as 100% in Kaduna state (Cluster A) to just 13% in Nassarawa state (Cluster C). This is consistent with the finding of an average 15% of general consumers being aware of aflatoxin in groundnut cake by Ezekiel *et al.* (2013). The levels of awareness in the Cluster C states of Bauchi and Nasarawa are substantially less than both current awareness levels in other Nigerian states and the levels of awareness in Ghana, Togo, and Benin in 2005 reported by James *et al.* (2007).

Product awareness is gradual, with some individuals within a group becoming aware sooner than others. Therefore, it is not surprising that some farmers are still not aware of aflatoxin in the two states of Kwara and Benue. Also, in a non-representative sample of eastern Ugandan groundnut farmers in 2014, 61% of household representatives knew of aflatoxin by name (Jelliffe *et al.*, 2016). An additional 31.5% of household representatives indicated hearing about ‘rotten nuts, mouldy, bitter taste,’ leading Jelliffe *et al.* (2016) to conclude that 92.5% of the sample group recognised aflatoxin as a problem in groundnut production. Therefore, more public awareness programs about the problems of aflatoxin are still needed in the region.

The rollout of Aflasafe was staggered through time with efforts from IITA and partners initially to test the efficacy of the product in hundreds of farmers’ fields and subsequently to incentivise the adoption of Aflasafe by the AgResults Nigeria pilot project. Kaduna state was among the first states where Aflasafe was field tested beginning in 2009 and then with the implementation of the AgResults pilot project starting in 2014. The steady growth of Aflasafe usage in Kaduna state illustrates significant adoption of this product. In contrast, Aflasafe usage started in Oyo state only in 2013 and the total number of users is much lower compared to Kaduna state. Among the farmers sampled in this study in Oyo state, the usage of Aflasafe increased in 2014 and then decreased. This may have been due to the common business challenges (financial and operational) faced by the agribusiness implementers during the initial stage of implementation of the AgResults pilot project in this state. It is important to note that this decrease is among the farmers who were surveyed and not necessarily the case for all farmers in Oyo State, and this may be due to sample selection. The differential performance of farmers across states could be due to a variety of factors including weather, credit and other business constraints. In addition, the performances of the different agribusiness firms participating in the implementation of the pilot project could also influence whether farmers continue the partnership and thus continue to use Aflasafe.

More of the surveyed farmers were repeat Aflasafe users. This is consistent with adoption theory of trial, followed by adoption or repeat use. It is expected that some users will renounce adoption for a variety of reasons. Some of them may also return and purchase the product in the future. Interestingly, persistence in Aflasafe usage was highest in Kaduna state where the average price of Aflasafe was the highest. Economic theory suggests that farmers facing higher prices are less likely to persist in using Aflasafe. However, the opposite was observed. This corresponds to the higher estimate of willingness to pay (WTP) for Aflasafe reported in Kaduna state (Ayedun *et al.*, 2017), which is consistent with the longer history of Aflasafe usage in the state and potentially farmers receiving price premiums for their Aflasafe-treated maize.

The coefficient on the Bundled dummy variable was positive and significant, indicating that farmers who purchase Aflasafe bundled with other inputs are more likely to persist in using the biocontrol product. Bundling Aflasafe with other inputs such as improved seeds, fertilisers and crop management practices may increase persistence because those inputs increase yield which in turn increase returns. During the 2014 season, average grain yield was 2.6 tons per hectare for farmers working with participating agribusiness firms in the AgResults pilot project compared to 1.7 tons per hectare as the average yield of maize in Nigeria (AgResults Initiative, 2017). Aflasafe does not have any effect on yield, so the improved yields in 2014 are attributed to improved input quality and improved crop management practices. It is important to note that in measuring whether Aflasafe was bundled with other inputs, the Bundled dummy variable may also be implicitly measuring the strength of participating agribusiness firm-farmer relationships.

Farmers with at least some formal education are more likely to persist in using Aflasafe. This result is consistent with the proposition earlier in the paper that farmers with formal education are more likely to understand the negative consequences of consuming aflatoxin-contained maize products. More educated farmers have previously been found to have a higher willingness to pay for Aflasafe in Nigeria (Ayedun *et al.,* 2017). This finding is also consistent with De Groote *et al.* (2016), who found that additional years of schooling increased WTP for verified, aflatoxinsafe maize. Similarly, Marechera and Ndwiga (2015) found that farmers with tertiary education were more likely to purchase commercial Aflasafe than farmers with no formal education.

The coefficient for the variable associated with whether the farmers perceive aflatoxin to stunt child growth was not statistically significant. This is most likely due to a lack of variability in the responses provided by the farmers. 88% of farmers who are aware of aflatoxin believe that eating aflatoxin-contaminated maize prevents children from growing fully. Similarly, the coefficient for the variable associated with the perception on price premium was not statistically significant. The result of this study may also have been driven by a lack of variation in the independent variable. 93% of those farmers who were aware of aflatoxin believe that aflatoxin-safe maize brings a price premium. The lack of significance of the Child Growth and Price Premium dummy variables does not mean that farmer perceptions of the impact of aflatoxin consumption on their families’ health and on their ability to sell aflatoxin-safe maize for a price premium have no impact on their behaviour. It just means that there was no statistical significance which can be due to lack of enough variability in the data, among other things. This analysis is suggesting factors that are influencing the repeat use of Aflasafe among surveyed farmers. However, more studies will be needed in this area to have a better understanding of the use of Aflasafe by farmers in Nigeria. Specifically, it will be important to have a better understanding of the role of expected or actual premium prices on farmers’ behaviour toward the use of Aflasafe in Nigeria.

## 5. Conclusions

Aflatoxin contamination has widespread, negative health and economic impacts on human and animal health in sub-Saharan Africa. This is particularly important for Nigeria, the second largest producer of maize in Africa. Consumption of safe and quality food is essential for economic growth and poverty alleviation. The biological control product, called Aflasafe, has substantial potential to mitigate these negative impacts. Adoption and persistent use of technologies such as Aflasafe that help improve quality in the food chain are important. This will require that farmers and the general public go through the stages of awareness of the aflatoxin problem, awareness of the potential solution, trial and finally adoption. The results reported here will guide development of approaches to enhance commercialisation and the adoption of Aflasafe leading to food security and income generation in Nigeria.

Expectedly, the level of awareness of the aflatoxin problem and the benefits of Aflasafe usage were linked to interventions—high in the states with interventions and low in the states without intervention. Furthermore, farmers who purchased Aflasafe bundled with other inputs were more likely to persist in using the product. Level of education had significant and positive impact on continued usage of Aflasafe. Continued interventions and general education of the public are recommended for increased awareness, trial and adoption of Aflasafe in Nigeria.
